# Personal and Neighborhood Socioeconomic Status as Determinants of Depression Outcomes among Older Adults

**DOI:** 10.1093/geroni/igaf122.248

**Published:** 2025-12-31

**Authors:** Yao Du, Stephanie Ming, Yin Wong, Dara Kiu, Yi Leung, Wai Chi Chan, Gloria Hoi, Yan Wong, Terry Lum

**Affiliations:** The University of Hong Kong, Hong Kong, China (People’s Republic); The University of Hong Kong, Hong Kong, China (People’s Republic); The University of Hong Kong, Hong Kong, China (People’s Republic); The University of Hong Kong, Hong Kong, China (People’s Republic); University of Reading, England, United Kingdom; The University of Hong Kong, Hong Kong, China (People’s Republic)

## Abstract

Background Disparities in mental health and their determinants in old age remain a critical yet understudied global issue. This study examines how individual and neighbourhood socioeconomic status (SES) interact to shape not only concurrent mental health but also treatment responses among older adults. Methods We recruited 4,381 older adults with subclinical depressive symptoms from the JC JoyAge programme, a large-scale community-based collaborative stepped-care intervention in Hong Kong, for this longitudinal analysis (2020–2023). Using multilevel modelling, we examined how individual SES (education, welfare status, housing type) and neighbourhood SES (disadvantage index) jointly affected depressive symptoms, anxiety symptoms, health-related quality of life (HRQoL), and treatment responses over time. Findings Neighbourhood disadvantage was linked to higher depressive symptoms and lower HRQoL at baseline (p < 0.01) and moderated treatment responses (p < 0.05). Cross-level interactions showed that individuals with low education and welfare reliance had higher depressive symptoms at baseline, while public housing residents had lower depressive symptoms, though these effects weakened in disadvantaged neighbourhoods (p < 0.05). Regarding treatment response, individuals with lower education and welfare reliance in disadvantaged areas had higher odds of meaningful mental health improvements (p < 0.05), whereas public housing residents had lower odds of meaningful clinical response in these areas (p = 0.07). Interpretations Findings reveal the nuanced interplay between individual and neighbourhood SES in mental health disparities. While some low-SES subgroups show resilience in disadvantaged environments, others remain vulnerable, emphasising the need for tailored, multilevel interventions that address structural barriers while strengthening individual coping resources.

